# Quantitative Analysis of Piezoresistive Characteristic Based on a P-type 4H-SiC Epitaxial Layer

**DOI:** 10.3390/mi10100629

**Published:** 2019-09-20

**Authors:** Yongwei Li, Ting Liang, Cheng Lei, Yingping Hong, Wangwang Li, Zhiqiang Li, Abdul Ghaffar, Qiang Li, Jijun Xiong

**Affiliations:** 1 Science and Technology on Electronic Test & Measurement Laboratory, North University of China, Taiyuan 030051, China; liyongwei27@163.com (Y.L.); liangtingnuc@163.com (T.L.); leicheng@nuc.edu.cn (C.L.); hongyingping0807@163.com (Y.H.); 18434365707@163.com (W.L.); lizhiqiangnuc@163.com (Z.L.); snjk08@163.com (Q.L.); 2 Department of Automation, Taiyuan Institute of Technology, Taiyuan 030051, China

**Keywords:** p-type 4H-SiC, piezoresistive effect, ohmic contact, cantilever beam

## Abstract

In this work, the piezoresistive properties of heavily doped p-type 4H-SiC at room temperature were investigated innovatively. It was verified by a field emission scanning electron microscope (FESEM), X-ray diffraction (XRD), and laser Raman spectroscopy (LRS) that the crystal quality of the epitaxial layer was good. The doping concentration and thickness of the epitaxial layer were measured by secondary ion mass spectrometry (SIMS) to be ~1.12 × 10^19^ cm^−3^ and ~1.1 µm, respectively. The 4H-SiC cantilever beam along $\left[1\overset{-}{1}00\right]$ crystal orientation was fabricated, and the fixed end of the cantilever beam was integrated with longitudinal and transverse p-type 4H-SiC piezoresistors. A good ohmic contact was formed between Ni/Ti/Al/Au and a p-type 4H-SiC piezoresistor under nitrogen environment annealing at 1050 °C for 5 min. The free end of the cantilever beam was forced to cause strain on the p-type 4H-SiC piezoresistor, and then the resistances were measured by a high precision multimeter. The experimental results illustrated that longitudinal and transverse gauge factors (GFs) of the p-type 4H-SiC piezoresistors were 26.7 and −21.5, respectively, within the strain range of 0–336*µε*. In order to further verify the electro-mechanical coupling effect of p-type 4H-SiC, the piezoresistors on the beam were connected to the Wheatstone full-bridge circuit and the output changes were observed under cyclic loading of 0–0.5 N. The measuring results revealed that the transducer based on the 4H-SiC piezoresistive effect exhibited good linearity and hysteresis, which confirmed that p-type 4H-SiC has the potential for pressure or acceleration sensing applications.

## 1. Introduction

Since the discovery of the semiconductor piezoresistive effect by Smith in 1954, the pressure sensors based on the piezoresistive effect have been the primary focus of many researchers, thanks to its simple reading circuit, stable performance, wide linear output range, easy IC integration, and many other advantages [1,2,3]. The most typical piezoresistive pressure sensor is based on the silicon piezoresistive effect, which electrically isolates the Si piezoresistor from the Si substrate by the PN junction. When the measured ambient temperature exceeds 120 °C, the PN junction reverses break down and the sensor performance is seriously degraded [4,5,6]. In order to achieve the pressure measurements in a higher temperature environment, a layer of silicon dioxide is introduced between the Si piezoresistor and the Si substrate, which is called the silicon-on-insulator (SOI). The SOI structure increases the operating temperature of the silicon piezoresistive pressure sensor to 400 °C, but in situ pressure measurement is still not possible for extreme environments, such as the drilling head, rocket engines, and aerospace, due to plastic deformation of silicon in high temperature environments exceeding 400 °C [7,8,9,10].

As one of the third-generation semiconductor materials, SiC has a large energy band gap, superior mechanical properties, and extreme chemical inertness. Accordingly, the SiC-based pressure sensors are considered to be the most promising semiconductor for use in extreme environments, such as high temperatures, strong corrosion, and high radiation [11,12,13]. Silicon carbide has more than 250 crystal types, while it has three major crystal types, such as 3C-, 4H-, and 6H- [14,15]. The 3C-SiC piezoresistive property has been experimentally confirmed with a gauge factor (GF) of about 30, and the GF was found to be stable at the temperatures ranging from 300 to 573 K [14]. However, 3C-SiC can only hetero-epitaxially grow on the Si substrate in the form of thin films, which limits the operating temperature and performance of the transducers-based 3C-SiC [16,17,18].

In contrast, α-SiC, such as 4H-SiC and 6H-SiC, can make all -SiC devices, which has attracted significant attention from many researchers [19,20,21]. So far, the research on the piezoresistive effect of 6H-SiC is relatively mature and the 6H-SiC pressure transducers capable of operating at 600 °C have been developed by Robert S. Okojie et al. from the NASA Glenn Research Center [22]. However, at 600 °C, the full-scale output of these transducers dropped by about 50–65% of the room temperature values, indicating that these devices have poor high temperature performance and cannot work at higher temperatures [22]. Therefore, in order to improve the performance of all-SiC pressure transducers, the piezoresistive effect of 4H-SiC has been studied in recent years [23,24,25]. In 2012, T Akiyama et al. [26] conducted the first study on the piezoresistive properties of n-type 4H-SiC with the longitudinal and transverse GFs of −10 and 20.8, respectively. Meanwhile, the piezoresistive properties of n-type 4H-SiC was found to be related to the size (length and width) of the resistance, which was not found in the piezoresistive effect of 6H-SiC [26]. In 2015, Robert S. Okojie et al. from the NASA Glenn Research Center demonstrated that n type 4H-SiC piezoresistive pressure sensors could operate at 800 °C and the characteristic sensitivity recovery beyond 400 °C reached values that were nearly equal to the room temperature values at 800 °C, and the anomaly may be caused by sensor encapsulation [27]. The above results have greatly encouraged scholars to study the piezoresistive properties of 4H-SiC further. Nguyen T. K. et al. experimentally verified the piezoresistive properties of moderate doped p-type 4H-SiC with transverse and longitudinal GF of −27.3 and 31.5, showing superiority over n-type 4H-SiC and 6 H-SiC [28]. However, to the best of our knowledge and literature review, research is needed to confirm the effect of heavy doped p-type 4H-SiC. These results and the piezoresistive effect of heavy doped p-type 4H-SiC has not been reported yet, which is focused on in this research.

In this paper, the p-type 4H-SiC epitaxial wafer was characterized by a field emission scanning electron microscope (FESEM), X-ray diffraction (XRD), laser Raman spectroscopy (LRS), and secondary ion mass spectrometry (SIMS) to verify the good quality of epitaxial layer crystal and obtain crucial electrical parameters, including doping concentration, epitaxial layer thickness, and so on. Reactive ion etching (RIE) was used to prepare SiC resistance strips. Through the thermally annealed process, a good ohmic contact was formed between p-type 4H-SiC and Ni/Ti/Al/Au. Cantilever beams with longitudinal and transverse p-type 4H-SiC piezoresistors along $\left[1\overset{-}{1}00\right]$ directions were prepared, which was used to measure the gauge factors at room temperature. The experimental results show that the heavy doped p-type 4H-SiC has the longitudinal and transverse GFs of 26.7 and −21.5, respectively. The four piezoresistors on a cantilever beam were then connected to be the Wheatstone full-bridge circuit, and the circuit output exhibited excellent linearity and hysteresis under cyclic loading of 0–0.5 N. These results demonstrate the application potential of p-type 4H-SiC in the mechanical sensing field.

## 2. Materials and Methods

### 2.1. Experimental Design

The phenomenon of the piezoresistive effect of semiconductor refers to the resistivity of semiconductor changes due to the change of carrier mobility when the semiconductor is subjected to stress. The gauge factor (GF) was introduced to quantify the piezoresistive effect of the semiconductor, which is defined as [29,30]:

(1) $$GF=\frac{△R/R}{\varepsilon }$$

where ε is the strain and △R/R is piezoresistor change rate with strain. Transverse piezoresistors have a current flow perpendicular to the direction of strain, and longitudinal piezoresistors have a current flow parallel to the direction of the strain, quantified using the transverse GF and longitudinal GF, respectively.

**Figure 1:**
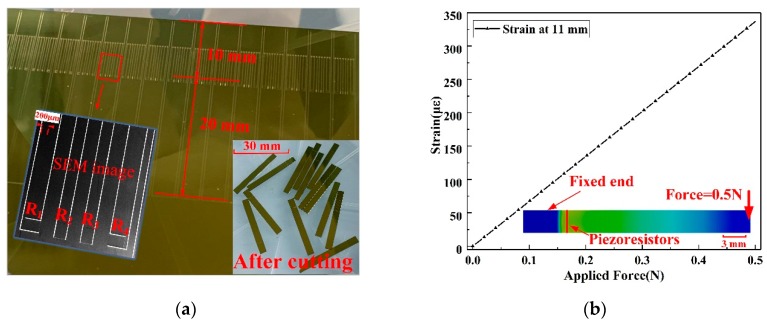
(**a**) Cantilever beam based on p-type 4H-SiC piezoresistors (*R*_1_, *R*_4_ longitudinal piezoresistors, *R*_2_, *R*_3_ transverse piezoresistors); (**b**) Finite element analysis (FEA) shows a linear gradient of the strain induced into the cantilever using the bending beam method.

In order to study the piezoresistive effect of p-type 4H-SiC, 4H-SiC cantilevers along $\left[1\overset{-}{1}00\right]$ directions were prepared with dimensions of 30-mm-long, 3-mm-wide, and 0.3-mm-thick, as illustrated in Figure 1a. The SiC cantilever was mounted at one end (0–10 mm) to a printed circuit board (PCB) using epoxy resin. Four p-type 4H-SiC piezoresistors with dimensions of 300 µm × 400 µm, including two transverse piezoresistors and two longitudinal piezoresistors, were prepared, centered at 11 mm from the fixed end of the cantilever. A known force (F) was applied to the free end of the cantilever, which induced strain (ε) at the upper surface of the beam and conducted it to the 4H-SiC piezoresistors. From beam mechanics, the strain (ε) induced into the 4H-SiC piezoresistor is given by [31,32,33,34]:

(2) $$\varepsilon =\frac{6F(l-x)}{Ebh^{2}}$$

where *E* is Young’s modulus of the 4H-SiC, *x* is the distance along the length of the cantilever measured from the root, *l* is the length, *b* is the width, and *h* is the thickness of the cantilever. When a force of 0–0.5 N was applied vertically to the free end of the cantilever, the strain at the center of piezoresistors was 0–336*µε*, which was consistent with ANSYS (Pittsburgh, PA, USA) simulation results, as shown in Figure 1b.

### 2.2. Characterization of the P-type 4H-SiC Epitaxial Layer

**Figure 2:**
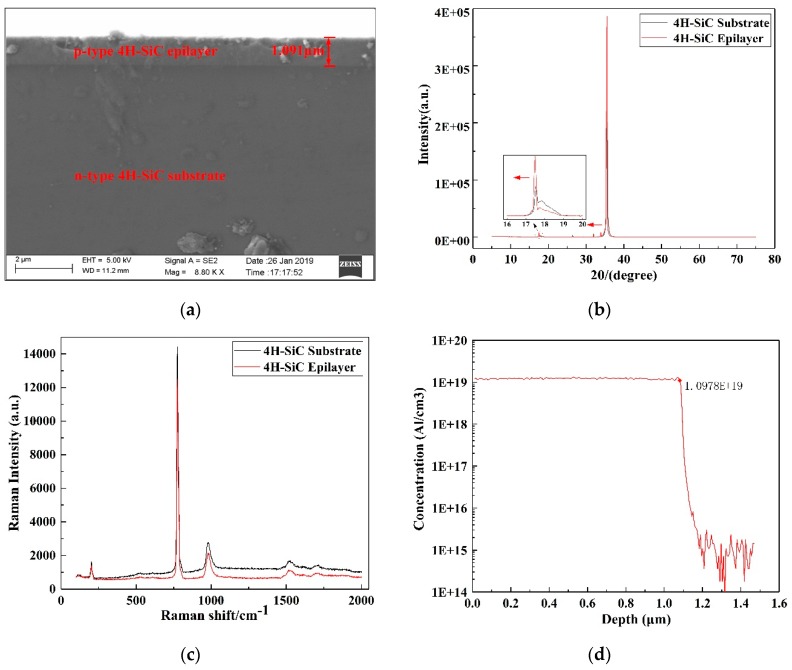
(**a**) Cross-sectional view of the p-type 4H-SiC epilayer on the n-type 4H-SiC substrate at 5000 times magnification. (**b**) X-ray diffraction (XRD) spectrum of the 4H-SiC epitaxial layer and substrate. (**c**) Raman spectra of the 4H-SiC epitaxial layer and substrate. (**d**) Secondary ion mass spectrometry (SIMS) profile of the 4H-SiC epitaxial layer.

The n-type 4H-SiC wafer (0001) used in this study was purchased from Tankeblue in China with a diameter of 100 mm, a thickness of 350 µm, a nitrogen doping concentration of 10^14^ cm^−3^, and Young’s modulus of 453 GPa. The n-type 4H-SiC buffer layer was homoepitaxially grown on an n-type 4H-SiC silicon surface with a nitrogen doping concentration of 10^18^ cm^−3^ and a thickness of 2 µm, followed by a p-type 4H-SiC piezoresistive layer with an aluminum doping concentration of 10^19^ cm^−3^ and thickness of 1 µm using the chemical vapor deposition (CVD). Characterization of the resulted 4H-SiC epitaxial wafer was done with the use of FESEM, XRD, LRS, and SIMS. Figure 2a highlights the illustrative SEM images of the cross-sectional view of the p-type 4H-SiC epilayer on the n-type 4H-SiC substrate at 5000 times magnification, which revealed the stratification phenomenon between the epilayer and substrate and the epitaxial thickness of ~1.091 µm. The use of XRD with Cu Kα radiation (*λ* = 1.5406) was made for the purpose to characterize the phase structure of the 4H-SiC epilayer. The XRD pattern (Figure 2b) revealed the fact that every characteristic peak of the 4H-SiC epilayer was substantially identical to the substrate and could be fully indexed as 4H-SiC, which certified that the epilayer belonged to just the 4C-SiC phase and had good crystallinity. Additionally, the characteristic peak of the epitaxial layer was slightly shifted to the left, which was attributable to dope aluminum atoms with a larger radius than carbon and silicon atoms, to make the lattice constant of SiC larger. The use of a laser Raman spectroscopy analyzer with an Ar^+^ atomic laser source (*P* = 100 mW, *λ* = 532 nm) was made for the purpose of characterizing the crystal quality of the 4H-SiC epitaxial layer. Figure 2c shows the Raman spectra of the n-type 4H-SiC substrate and the p-type 4H-SiC epitaxial layer. As can be seen, the peaks of the two Raman spectra are very close and consistent with the standard 4H-SiC Raman spectroscopy data, indicating that the epitaxial layer continues the crystal type of the substrate well and possesses good crystalline quality. The difference between the two Raman spectra may be due to the type and concentration of doping elements. For the characterization of actual doping parameters, the SIMS was employed to monitor the 4H-SiC epitaxial layer. The SIMS profile (Figure 2d) reveals the fact that the doping concentration of Al element is uniform up to ~1.1 × 10^19^ cm^−3^ and the doping thickness is ~1.1 µm.

### 2.3. Fabrication of 4H-SiC Cantilever Beam

**Figure 3:**
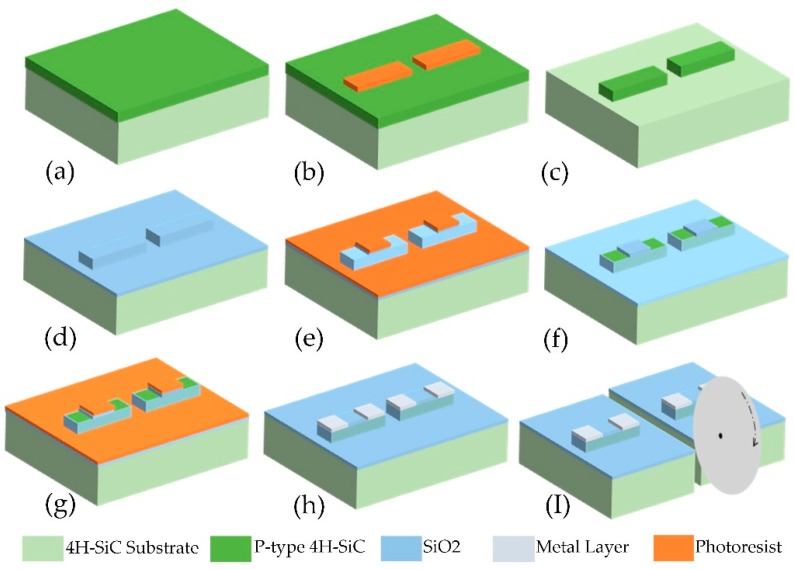
The processing flow of 4H-SiC cantilever beam structure. (**a**) P-type 4H-SiC epitaxial wafer. (**b**) Piezoresistive structures were patterned by the first lithography. (**c**) Resistance stripes were etched using reactive ion etching (RIE). (**d**) The SiO_2_ isolation layer was grown by plasma-enhanced chemical vapor deposition (PECVD). (**e**) The ohmic contact hole was patterned by the second lithography. (**f**) The ohmic contact hole was opened in buffered HF solution. (**g**) Metal structures were patterned by the third lithography. (**h**) Ni 200 Å/Ti 500 Å/Al 3000 Å/Au 3000 Å were deposited and then patterned by stripping the AZ6130 photoresist in acetone. (**I**) The cantilever beam structures were prepared by using an automatic dicing saw.

The cantilever beams for characterizing the p-type 4H-SiC piezoresistive effect were prepared using a standard micro-electro-mechanical systems (MEMS) process, as shown in Figure 3. The fabrication process of the cantilever includes the following primary steps: Piezoresistive etching, metal contact hole machining, metal leads deposition, and so on.

**Figure 4:**
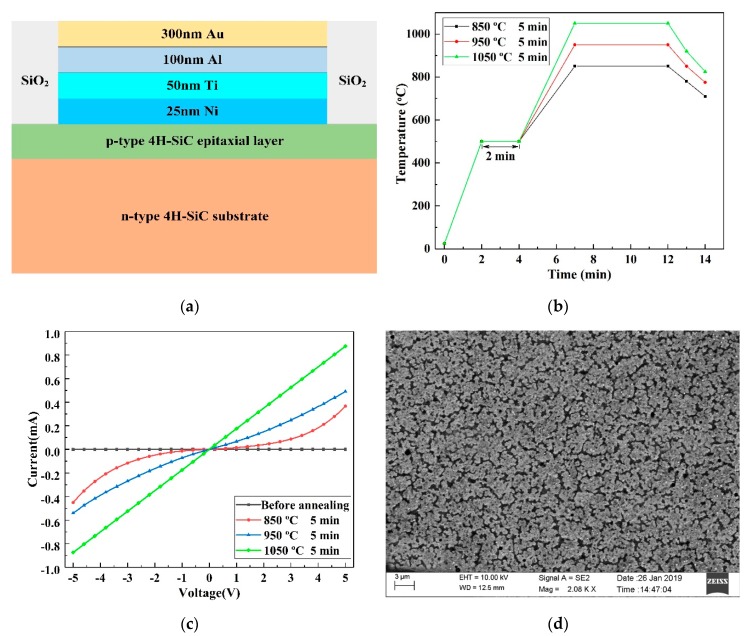
(**a**) The metal hierarchy inside the ohmic contact hole. (**b**) Temperature profile of the annealing process. (**c**) Current–voltage characteristics of the Ni/Ti/Al/Au and p-type 4H-SiC contact before and after annealing. (**d**) SEM image of the metal surface morphology after annealing at 1050 °C.

Firstly, the 4H-SiC wafer was cleaned using the root cause analysis (RCAprocess. The wafer was then dry-oxidized at 1100 °C for 4 h in a thermal oxidation furnace, and the thickness of the SiO_2_ film was measured for 110 nm using NANOMETRICS Nanospec/AFT 210 (Milpitas, CA, USA), after which the oxide was corroded away in buffered HF solution, rinsed in de-ionized (DI) water and blow dried in nitrogen. The above procedure typically removes suspended Si- and C- bonds and chlorine, which resides on the surface, to leave a cleaner surface. After the first lithographic process, the epilayer was dry-etched to form a piezoresistor by reactive ion etching (RIE) with a 5.3 µm AZ4620 photoresist mask. The etching parameters were as follows: Chamber pressure of 2 Pa, power of 200 W, and SF6 with a flow rate of 50 sccm. After etching for 11 min, the height of the piezoresistive mesa was measured to 1.3 µm using the step profiler, which was more significant than the thickness of the p-type 4H-SiC epitaxial layer and ensured the piezoresistor was insulated from the substrate. Subsequently, to insulate the metal leads from SiC substrates, a 200 nm SiO_2_ thin film was deposited on the silicon surface of the 4H-SiC wafer by plasma-enhanced chemical vapor deposition (PECVD). Next, the second lithographic process was performed to pattern the metal contact holes, which were opened in buffered HF solution with a 2.3 µm AZ6130 photoresist mask. After the third lithographic process, Ni 250 Å/Ti 500 Å/Al 1000 Å/Au 3000 Å (Figure 4a) was deposited on the photoresist mask by magnetron sputtering and were defined by stripping the AZ6130 photoresist in acetone. Further, the wafer was diced into rectangular beams for varistor characterization by DISCO DAD322 (an automatic dicing saw) (Disco Corporation, Tokyo, Japan). Finally, these beams were heat-treated using the GSL-1100X-RTP50 (HF-Kejing, Hefei, China), a rapid heat treatment furnace, to achieve excellent ohmic contacts between the metal and p-type 4H-SiC.

## 3. Results and Discussion

A total of 10 identical SiC cantilever structures were manufactured in the same batch and showed the same piezoresistive effect in the experiments described below. In order to form a good ohmic contact on SiC, it is essential to anneal at temperatures exceeding 800 °C. After annealing under a nitrogen environment at different temperatures, as shown in Figure 4b, Keithley 4200 semiconductor analyzers were used to measure the relationship between current (I) and voltage (V) of SiC piezoresistors. Figure 4c shows the I/V characteristics of the piezoresistor before and after annealing, which indicates that good ohmic contact has been formed between Ni/Ti/Al/Au and p-type 4H-SiC after annealing under a nitrogen environment at 1050 °C for 5 min. Based on the test results, it can be calculated that the sheet resistances of the 1.1 µm p-type layer is 5.6 KΩ/□. In addition, the metal’s surface morphology was characterized by SEM after annealing at 1050 °C, as shown in Figure 4d. It can be seen that the ultra-high annealing temperature only slightly affects the surface compactness, which did not affect the electrical test of the SiC piezoresistive.

**Figure 5:**
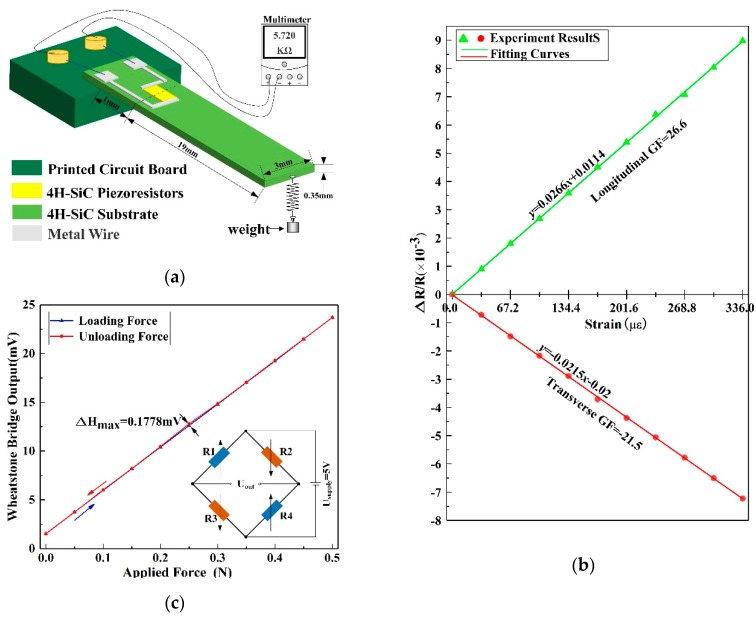
(**a**) Test structure of the p-type 4H-SiC piezoresistive effect. (**b**) The relative change in resistance (Δ*R*/*R*) of the beams as a function of the strain. (**c**) Output voltage of the Wheatstone bridge as a function of applied load at room temperature (*R*_1_, *R*_2_, *R*_3_, and *R*_4_ are SiC piezoresistors on the beam, as shown in Figure 1a).

Next, a force of 0–0.5 N with the increment of 0.05 N was applied to the fixed end of the assembled cantilever beam and at the same time the resistances of p-type 4H-SiC piezoresistors were measured by a high precision multimeter, as shown in Figure 5a. Figure 5b shows the change rate of SiC piezoresistor (△*R/R*) functions with the strain caused by the external force. By fitting the test results, the longitudinal and transverse GFs of p-type 4H-SiC piezoresistives are 26.7 and −21.5, respectively, which are comparable to the previous results in the reported literature [28] and have larger gauge factors (GFs) than 6H-SiC and n-type 4H-SiC [26,35] under the condition of heavy doping. In order to further verify the electro-mechanical coupling effect of SiC, the piezoresistors on the beam were placed into a four-arm Wheatstone bridge, and a fixed input voltage of 5 V was applied. The output of the bridge is a function of the cantilever deflection under cyclic loading of 0–0.5 N, as shown in Figure 5c. The relationship between the SiC piezoresistors’ change and the measured output voltage (*V_out_*) is:

(3) $$V_{out}=\frac{V_{in}}{2}\left(\;\frac{R_{1}-R_{3}}{R_{1}+R_{1}}-\frac{R_{4}-R_{2}}{R_{1}+R_{1}}\;\right)$$

where *V_in_* = 5 V is the supplied voltage of the Wheatstone bridge and *R*_1_, *R*_2_, *R*_3_, and *R*_4_ are SiC piezoresistors on the beam, as shown in Figure 1a. It can be seen that the piezoresistance of p-type 4H-SiC exhibited good linearity and low hysteresis. The above experiments have proved that p-type 4H-SiC has a good piezoresistive effect, which demonstrates the application potential of p-type 4H-SiC in the mechanical sensing field.

## 4. Conclusions

The piezoresistive effect of heavily doped p-type 4H-SiC was characterized experimentally by applying the cantilever beam. It has been shown that longitudinal and transverse GFs of 4H-SiC piezoresistives are 26.7 and −21.5 respectively. Moreover, transducers based on the 4H-SiC piezoresistive effect was proved to have good linearity and hysteresis, which indicated that p-type 4H-SiC could be used to measure the change of mechanical parameters, such as pressure, acceleration, strain, and flow.
